# Inhibitory Effect of Tangeretin and Cardamonin on Human Intestinal SGLT1 Activity In Vitro and Blood Glucose Levels in Mice In Vivo

**DOI:** 10.3390/nu13103382

**Published:** 2021-09-26

**Authors:** Hideo Satsu, Ryosuke Shibata, Hiroto Suzuki, Shimon Kimura, Makoto Shimizu

**Affiliations:** 1 Department of Biotechnology, Faculty of Engineering, Maebashi Institute of Technology, Gunma 371-0816, Japan; h.suzuki.1306@gmail.com (H.S.); m2166006@maebashi-it.ac.jp (S.K.); 2 Department of Applied Biological Chemistry, Graduate School of Agricultural and Life Sciences, The University of Tokyo, Tokyo 113-8657, Japan; ryosuke.shibata.1016@gmail.com; 3 Department of Nutritional Science, Tokyo University of Agriculture, Tokyo 156-8502, Japan; ms205346@nodai.ac.jp

**Keywords:** SGLT1, transporter, tangeretin, cardamonin, intestinal epithelial cell

## Abstract

Rapid postprandial blood glucose elevation can cause lifestyle-related diseases, such as type II diabetes. The absorption of food-derived glucose is primarily mediated by sodium/glucose cotransporter 1 (SGLT1). Moderate SGLT1 inhibition can help attenuate postprandial blood glucose elevation and prevent lifestyle-related diseases. In this study, we established a CHO cell line stably expressing human SGLT1 and examined the effects of phytochemicals on SGLT1 activity. Among the 50 phytochemicals assessed, tangeretin and cardamonin inhibited SGLT1 activity. Tangeretin and cardamonin did not affect the uptake of L-leucine, L-glutamate, and glycyl-sarcosine. Tangeretin, but not cardamonin, inhibited fructose uptake, suggesting that the inhibitory effect of tangeretin was specific to the monosaccharide transporter, whereas that of cardamonin was specific to SGLT1. Kinetic analysis suggested that the suppression of SGLT1 activity by tangeretin was associated with a reduction in V_max_ and an increase in K_m_, whereas suppression by cardamonin was associated with a reduction in V_max_ and no change in K_m_. Oral glucose tolerance tests in mice showed that tangeretin and cardamonin significantly suppressed the rapid increase in blood glucose levels. In conclusion, tangeretin and cardamonin were shown to inhibit SGLT1 activity in vitro and lower blood glucose level in vivo.

## 1. Introduction

Diabetes is a lifestyle-related disease with increasing global incidence. According to the reports of the International Diabetes Federation, in 2019, the number of patients with diabetes was approximately 463 million worldwide, and by 2045, it is predicted to increase to approximately 700 million [1]. Diabetes is classified as insulin-dependent type I and insulin-non-dependent type II, and approximately 95% of patients with diabetes are diagnosed with type II diabetes. In addition to genetic factors, environmental factors such as excessive nutrient intake and lack of physical activity are important contributors to the onset of type II diabetes. Chronic hyperglycemia, insulin resistance, and obesity are typical characteristics of patients with type II diabetes, which further causes complications such as retinopathy, nephropathy, and neuropathy [2,3].

The excessive intake of carbohydrates, particularly saccharides, is a leading cause of the increasing incidence of type II diabetes and obesity. α-amylase digests saccharides consumed with meals to oligosaccharides, which are further digested to monosaccharides by α-glucosidases, such as sucrase-isomaltase or maltase, in the intestinal tract. In intestinal epithelial cells, glucose is primarily absorbed by sodium-dependent glucose transporter 1 (SGLT1), expressed at the apical membrane of intestinal epithelial cells [4,5]. The incorporated glucose is transported to the blood via glucose transporter 2 (GLUT2), which is expressed at the basolateral membrane of intestinal epithelial cells. Recently, it has been reported that GLUT2 present in cells is translocated to the apical membrane and contributes to glucose uptake under high glucose concentrations [6,7,8].

To prevent diabetes, it is advisable to inhibit α-amylase or α-glucosidase activity, or to suppress SGLT1 activity in moderation, by using food components to reduce rapid spikes in blood glucose levels after meals and prevent chronic hyperglycemia. Several food substances, such as wheat albumin [9,10] and guava tea polyphenols [11], have been reported to inhibit α-amylase or α-glucosidase activity, which helps suppress rapid spikes in blood glucose levels after meals.

Intestinal epithelial cells play an important role in the absorption of nutrients and functional components from food. Furthermore, since intestinal epithelial cells are exposed to food components at the highest concentration and frequency, their functions are considered to be controlled and regulated by food components. We previously reported that the functions of intestinal epithelial cells, such as transporter activity, cytokine secretion, and gene expression of detoxification enzymes, are regulated or modulated by various food components [12,13]. Among intestinal transporters, the taurine transporter was shown to be suppressed by sesame extracts, with lysophosphatidylcholine being one of the inhibitory compounds [14,15]. The intestinal fructose transporter (GLUT5) is also suppressed by flavonoids [16]. Furthermore, MDR1 activity was inhibited by 2-monopalmitin [17]. These studies were mostly performed using in vitro colon adenocarcinoma-derived intestinal epithelial-like Caco-2 cells. However, it is difficult to assess SGLT1 activity using these cell lines, as Caco-2 cells have often been reported to exhibit extremely low or no SGLT1 activity. Steffansen et al. [18,19] also reported that SGLT1-mediated transport is highly dependent on the cell bank’s origin. Therefore, a simple in vitro evaluation system to assess SGLT1 activity is necessary to exhaustively identify food components that can inhibit SGLT1 activity.

In the present study, we constructed a stable human SGLT1-expresing CHO cell line and used this evaluation system to search for and analyze food components, especially phytochemicals, which inhibit SGLT1 activity. To further evaluate the suppressive effect of phytochemicals on SGLT1 activity in vivo, the effect of phytochemicals on glucose absorption in mice was examined using oral glucose tolerance tests (OGTTs).

## 2. Materials and Methods

### 2.1. Materials

The following materials were purchased from the listed sources: CHO cell line and Caco-2 cells from the American Type Culture Collection (Rockville, MD, USA); Dulbecco’s modified Eagle’s medium (DMEM) containing 4 mM L-glutamine from Wako Chemicals (Osaka, Japan); penicillin–streptomycin (10,000 U/mL and 10 mg/mL in 0.9% sodium chloride, respectively) from Gibco (Gaithersburg, MD, USA); fetal calf serum (FCS) from Life Technologies (Grand Island, NY, USA); non-essential amino acids (NEAA) from Cosmobio (Tokyo, Japan); [^3^H]-glucose (specific radioactivity: 21.2 Ci/mmol), [^3^H]-fructose (5.0 Ci/mmol), [^3^H]-L-leucine (142 Ci/mmol) from GE Healthcare (Fairfield, CT, USA), [^3^H]-L-glutamic acid (46 Ci/mmol), and [^3^H]-glycyl-sarcosine ([^3^H]-Gly-Sar) (0.2 Ci/mmol) from Moravek (Brea, CA, USA); and a type-I collagen solution from Nitta Gelatin (Osaka, Japan). The phytochemicals used for this study are shown in Table A1. All other chemicals used were of reagent grade.

### 2.2. Transfection of Human SGLT1 Expression Vectors for Development of a Stable Cell Line

Chinese hamster ovary-K1 (CHO-K1) cells were cultured in Ham’s F-12 medium supplemented with 10% FCS and penicillin–streptomycin. A human SGLT1 expression vector (pcDNA3.1-SGLT1) was constructed as follows: the open reading frame of human SGLT1 was amplified from a plasmid encoding human SGLT1 (Flexi clone, Promega) using the primer 5’-GGGAAGCTTATGGACAGTAGCACCTGGAG-3’, which introduced a *Hind*III site, and the primer 5’-GGGGAATTCGGCAAAATATGCATGGCAAAAG-3’, which introduced an *Eco*RI site. The *Hind*III/*Eco*RI-digested PCR fragment was ligated into the digested pcDNA3.1A (Invitrogen, Carlsbad, CA, USA), thereby creating pcDNA-hSGLT1, and sequenced. 

The plasmid vector was transfected into CHO-K1 cells using Lipofectamine 2000 (Invitrogen) according to the manufacturer’s instructions. hSGLT1-expressing cells (transfected cells) were selected with G418, and limiting dilution was performed in 96-well plates. Several single clones were selected from the 96-well plates. The clone that exhibited the strongest sodium-dependent [^3^H]-glucose uptake activity was selected and the hSGLT1 stable cell line was established.

### 2.3. Assay of Glucose Uptake in a Stable Cell Line Expressing hSGLT1

For the glucose uptake assay, the cells were seeded in a 24-well plate at 1.0 × 10^5^ cells per well in the Ham’s F-12 medium supplemented with 10% FCS, 100 U/mL penicillin, 100 µg/mL streptomycin, and 2 mg/mL G418. After seeding for two days, the glucose uptake experiment was performed. The cells were washed twice with 700 μL of phosphate-buffered saline (PBS) and incubated once in 300 μL of uptake buffer (140 mM NaCl, 0.34 mM Na_2_HPO_4_, 0.44 mM KH_2_PO_4_, 5.33 mM KCl, 1.26 mM CaCl_2_, 0.49 mM MgCl_2_, 0.41 mM MgSO_4_, and 4.16 mM NaHCO_3_; pH adjusted to 7.4 with KOH) for 10 min. The cells were then incubated with 50 nM [^3^H]-glucose in 300 μL of the uptake buffer, without or with each phytochemical at 37 °C for 10 min. After the incubation, the buffer was removed, and the cells in each well were carefully washed three times with 700 μL of ice-cold PBS containing 0.05% sodium azide. Subsequently, 250 μL of 0.1% Triton X-100 was added to each well, following which the lysed cells were collected in 3 mL of a scintillation cocktail, and the tritium content of the cells from each well was measured using an LSC 5100 liquid scintillation analyzer (Aloka, Tokyo, Japan).

### 2.4. Caco-2 Cell Culture

Caco-2 cells were cultured in plastic dishes of 78.5 cm^2^ containing a culture medium composed of DMEM, 10% FCS, 1% NEAA, 2% glutamine, 100 U/mL penicillin, and 100 μg/mL streptomycin. The cells were incubated at 37 °C in a humidified atmosphere of 5% CO_2_ in air, and the culture medium was replaced at a split ratio of 4:8 by trypsinization with a solution containing 0.1% trypsin and 0.02% EDTA in PBS. For uptake experiments, Caco-2 cells were seeded in a 24-well plate at 1.0 × 10^5^ cells per well. After 14 days of culture, the [^3^H]-fructose or [^3^H]-Gly-Sar uptake by Caco-2 cells was measured as previously described [16].

### 2.5. OGTTs in Mice

The animal care procedures and methods adopted were approved by the Animal Care and Use Committee of The University of Tokyo (permission number: P13-840).

ICR mice (6–8 weeks old, male, purchased from CLEA Japan, Japan) were fasted overnight for 18 hours. The tangeretin or cardamonin was suspended in an 0.3% *w*/*v* solution of carboxymethyl cellulose sodium salt, respectively. The mice were orally administered the vehicle or phytochemical (tangeretin or cardamonin) at 250 or 400 mg/kg body weight (BW). The mice were orally administered a 20% glucose solution (1 g/kg BW). Blood drawn from the orbital venous sinus under isoflurane anesthesia at 30, 60, and 120 min after the glucose’s administration was collected in heparinized tubes. The plasma glucose concentration was measured using the glucose CII-test WAKO according to the manufacturer’s instructions (WAKO, Osaka, Japan). The area under the curve (AUC) was calculated using the trapezoidal rule.

### 2.6. Statistical Analysis

Data are expressed as the mean ± standard error of the mean from at least three independent experiments performed in triplicate. Statistical comparisons were performed using Student’s *t*-test, Dunnett’s test, or Tukey’s test. Differences were considered statistically significant at *p* < 0.05.

## 3. Results

### 3.1. Construction of the Stable hSGLT1-Expressed Cell Line

Ninety-four G418-resitant clones in 96-well plates were passed to 48-well plates and then to 24-well plates. After confluence was achieved in the 24-well plates, the glucose uptake activity of each cell was measured. Among 91 clones, 10 clones that exhibited high glucose uptake activity were selected, and glucose uptake in the selected 10 clones was measured in the absence or presence of sodium ions. Among the 10 clones, the clone with the highest sodium-dependent glucose uptake (4G8) was selected as the hSGLT1 clone. We confirmed that glucose uptake in the 4G8 clone was significantly suppressed by phlorizin, a typical SGLT inhibitor, suggesting that glucose uptake in the 4G8 clone occurred via SGLT1. Therefore, the 4G8 clone was used in the subsequent experiments.

### 3.2. Characterization of Sodium-Dependent Glucose Uptake Activity in Stable hSGLT1-Expressing Cells

**Figure 1:**
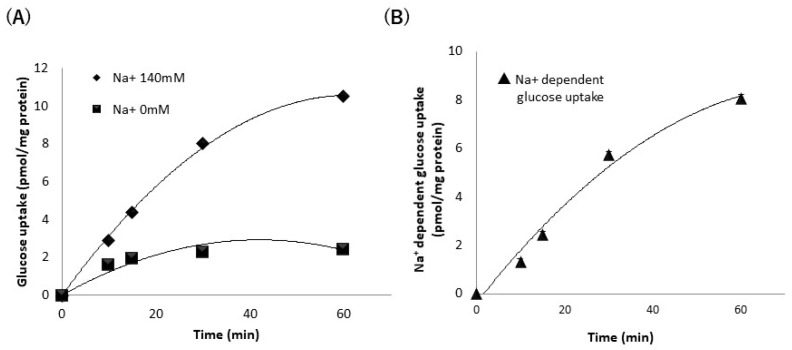
Glucose uptake in stable hSGLT1-expressing Chinese Hamster Ovary (CHO) cells in the absence or presence of sodium ions (**A**). (**B**) Sodium-dependent glucose uptake in stable hSGLT1-expressing CHO cells. Glucose uptake in the absence or presence of sodium ions was measured (**A**). Sodium-dependent glucose uptake was calculated by subtracting the glucose uptake in the presence of sodium ions and in the absence of sodium ions. Each value represents the mean ± standard error of mean (*n* = 3).

To determine the optimal conditions for assessing SGLT1 activity, the glucose uptake activity was measured in the absence or presence of sodium ions at different incubation times. Figure 1A shows that glucose uptake in the absence or presence of sodium ions increased in a time-dependent manner. Sodium-dependent glucose uptake also increased in a time-dependent manner (Figure 1B). Based on this result, we fixed the incubation time at 30 min to assess sodium-dependent glucose uptake (SGLT1) activity, as SGLT1 activity was greater than sodium-independent glucose uptake activity.

### 3.3. Effect of Phytochemicals on Glucose Uptake in hSGLT1-Expressing CHO Cell Line

**Figure 2:**
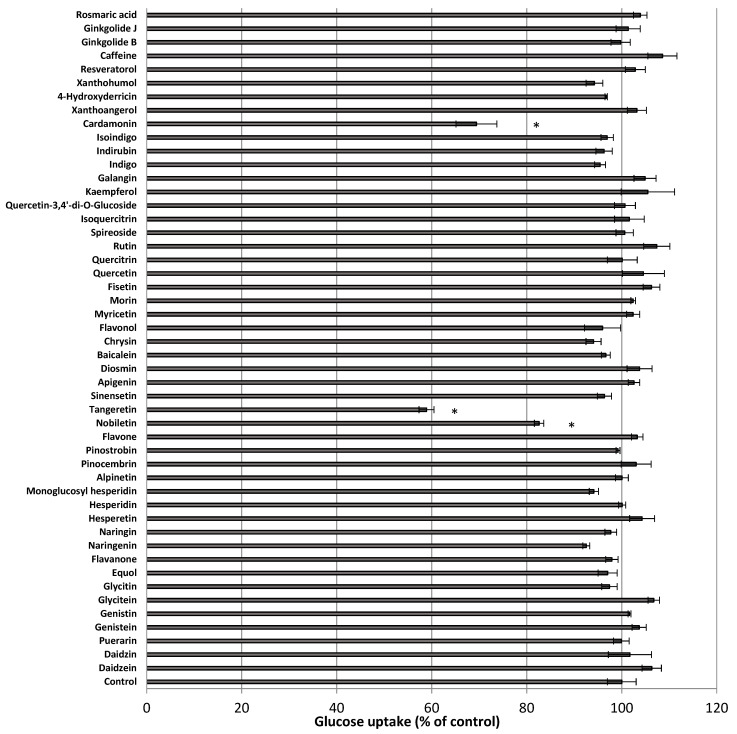
Effect of the phytochemicals on glucose uptake in stable hSGLT1-expressing CHO cells. Glucose uptake was measured in the absence or presence of the phytochemicals (1 μM). The values are expressed in terms of mean ± standard error of mean (*n* = 3); * *p* < 0.05 vs. the control value (Student’s *t*-test).

**Figure 3:**
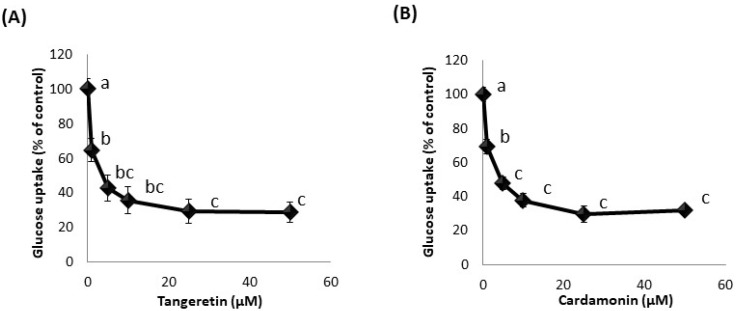
Concentration-dependent inhibition of glucose uptake by tangeretin (**A**) and cardamonin (**B**) in stably hSGLT1-expressed CHO cells. Glucose uptake was measured in the absence or presence of 0–50 μM tangeretin (**A**) and cardamonin (**B**). The values are the mean ± SE (*n* = 3), and the values indicated by different characters are significantly different from each other (Tukey’s test; *p* < 0.05).

Using the developed hSGLT1-expressing CHO cell line, we attempted to identify phytochemicals that inhibit glucose uptake activity. Among about 50 types of phytochemicals, we found that cardamonin, tangeretin, and nobiletin significantly inhibited glucose uptake in hSGLT1-expressing CHO cells (Figure 2). Among these phytochemicals, we focused on tangeretin and cardamonin, as tangeretin and nobiletin have similar structures, and tangeretin exhibited greater inhibitory activity than nobiletin. Next, we examined the effect of these phytochemicals on glucose uptake when administered at various concentrations (0–50 μM). Figure 3 clearly shows that glucose uptake was inhibited in a dose-dependent manner by tangeretin (Figure 3A) and cardamonin (Figure 3B).

### 3.4. Effect of Tangeretin and Cardamonin on Nutrient Uptake in hSGLT1/CHO Cells and Human Intestinal-like Caco-2 Cells

**Figure 4:**
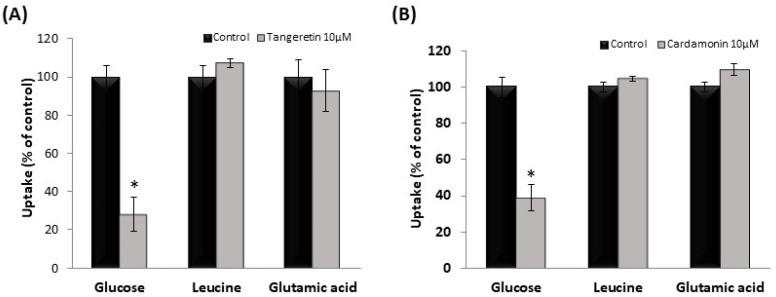
Effect of tangeretin (**A**) and cardamonin (**B**) on leucine and glutamic acid uptake in stable hSGLT1-expressing CHO cells. The uptake of glucose, L-leucine, and L-glutamic acid uptake was measured in the absence or presence of 10 μM tangeretin (**A**) or cardamonin (**B**). The values are expressed in terms of mean ± standard error of mean (*n* = 3); * *p* < 0.05 vs. the control value (Student’s *t*-test).

We examined the effect of tangeretin and cardamonin on the uptake of [^3^H]-glucose (50 nM), [^3^H]-L-leucine (6.25 nM), and [^3^H]-L-glutamic acid (22.2 nM). At 10 μM, tangeretin and cardamonin did not affect the uptake of L-leucine or L-glutamic acid, but suppressed glucose uptake (Figure 4A,B). These results suggested that the phytochemicals exerted no effect on amino acid transporters involved in L-leucine or L-glutamic acid uptake.

**Figure 5:**
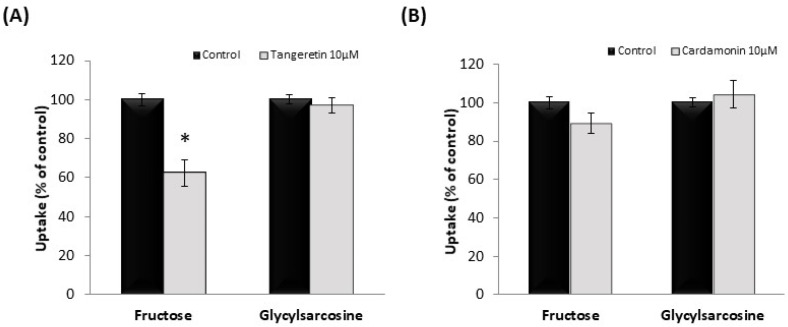
Effect of tangeretin (**A**) and cardamonin (**B**) on fructose and glycyl-sarcosine uptake in Caco-2 cells. Fructose and glycyl-sarcosine uptake were measured in the absence or presence of 10 μM tangeretin (**A**) or cardamonin (**B**). The values are expressed in terms of mean ± standard error of mean (*n* = 3); * *p* < 0.05 vs. the control value (Student’s *t*-test).

Caco-2 cells are often used as an in vitro model for human intestinal epithelial cells and express fructose transporter (GLUT5) and di- and tri-peptide transporter (PepT1). Therefore, we investigated the effects of treatment with the phytochemicals on the uptake of fructose and Gly-Sar, a typical substrate of PepT1. As shown in Figure 5A, tangeretin significantly inhibited fructose uptake, but not Gly-Sar uptake. Conversely, cardamonin did not inhibit fructose or Gly-Sar uptake (Figure 5B).

### 3.5. Effect of Tangeretin-Related Compounds on Glucose Uptake in hSGLT1/CHO Cells

**Figure 6:**
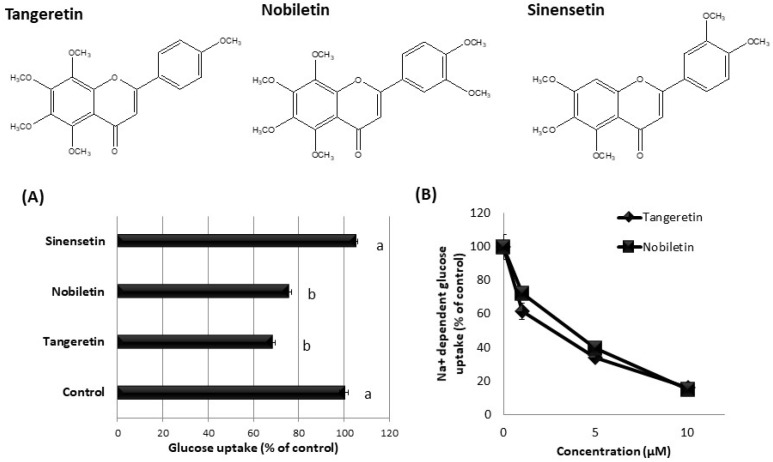
Effect of tangeretin-related compounds on glucose uptake in stable hSGLT1-expressing CHO cells. Glucose uptake was measured in the absence or presence of 1 μM tangeretin, nobiletin, and sinensetin (**A**). The values are expressed in terms of mean ± standard error of mean (SE) (*n* = 3), and the values indicated by the different characters are significantly different from each other (Tukey’s test; *p* < 0.05). Sodium-dependent glucose uptake was assessed by subtracting glucose uptake in the presence of sodium ions and in the absence of sodium ions, further, in the absence or presence of tangeretin-related compounds (**B**). Each value represents mean ± SE (*n* = 3).

We investigated the inhibitory effect of tangeretin-related compounds on glucose uptake in hSGLT1-expressing cells. As shown in Figure 6A, tangeretin and nobiletin significantly inhibited the glucose uptake, whereas sinensetin did not affect the glucose uptake in hSGLT1/CHO cells. Tangeretin and nobiletin dose-dependently inhibited sodium-dependent glucose uptake (Figure 6B), suggesting that these phytochemicals inhibit SGLT1 activity.

### 3.6. Effect of Cardamonin-Related Compounds on Glucose Uptake in hSGLT1/CHO Cells

**Figure 7:**
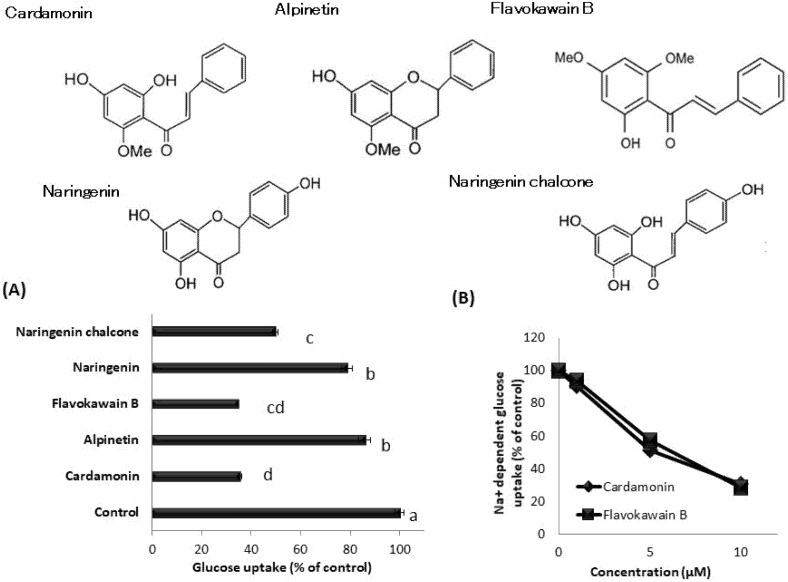
Effect of cardamonin-related compounds on glucose uptake in stable hSGLT1-expressing CHO cells. Glucose uptake was measured in the absence or presence of 10 μM cardamonin and its related compound, respectively (**A**). The values represent the mean ± standard error of mean (SE) (*n* = 3), and the values indicated by different characters are significantly different from each other (Tukey’s test; *p* < 0.05). Sodium-dependent glucose uptake was assessed by subtracting glucose uptake in the presence of sodium ions and in the absence of sodium ions, and further, in the absence or presence of cardamonin-related compounds (**B**). Each value represents the mean ± SE (*n* = 3).

We also investigated the inhibitory effect of cardamonin-related compounds on the glucose uptake in hSGLT1-expressed cells. Figure 7A shows that flavokawain B, naringenin chalcone, and cardamonin markedly inhibited glucose uptake. Naringenin and alpinetin also significantly inhibited glucose uptake, but to a lesser extent. To confirm whether cardamonin inhibits SGLT1 activity, we evaluated the effect of cardamonin and related compounds on sodium-dependent glucose uptake. Cardamonin and flavokawain B inhibited sodium-dependent glucose uptake in a dose-dependent manner (Figure 7B), suggesting that these phytochemicals inhibit SGLT1 activity.

### 3.7. Kinetic Analysis of Sodium-Dependent Glucose Uptake by hSGLT1/CHO Cells in the Absence or Presence of Tangeretin and Cardamonin

**Figure 8:**
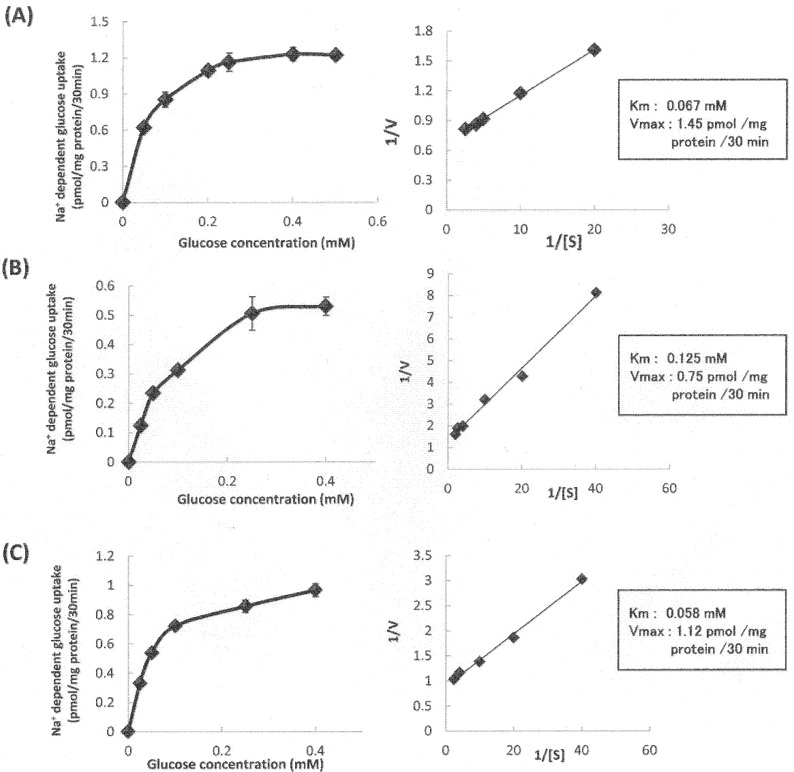
Kinetic analysis of sodium-dependent glucose uptake in the absence (**A**) or presence of tangeretin (**B**) and cardamonin (**C**). Sodium-dependent glucose uptake was measured after the administration of 0–0.4 mM glucose without (**A**) or along with 10 μM tangeretin (**B**) or cardamonin (**C**). The values are expressed in terms of mean ± standard error of mean (*n* = 3). Lineweaver–Burk plots were constructed to calculate the Vmax and Km values for sodium-dependent glucose uptake in the absence or presence of 10 μM tangeretin or cardamonin.

A kinetic analysis of SGLT1 activity was performed, and Lineweaver–Burk plots were constructed to calculate the maximal velocity (V_max_) and K_m_ values for glucose uptake in cells in the absence (Figure 8A) or presence of 10 μM tangeretin (Figure 8B) and cardamonin (Figure 8C). The V_max_ values in the presence of tangeretin and cardamonin decreased compared to that of control cells. However, the K_m_ values were not significantly different between the control and cardamonin-treated groups, although the K_m_ value increased upon tangeretin treatment. These results suggest that the inhibition of SGLT1 activity was primarily attributed to the decrease in the V_max_ value in the case of treatment with cardamonin, and to the decrease in the V_max_ value and increase in the K_m_ value in the case of treatment of tangeretin.

### 3.8. Effect of Tangeretin and Cardamonin on the Increase in Blood Glucose Levels In Vivo Following Oral Glucose Administration

**Figure 9:**
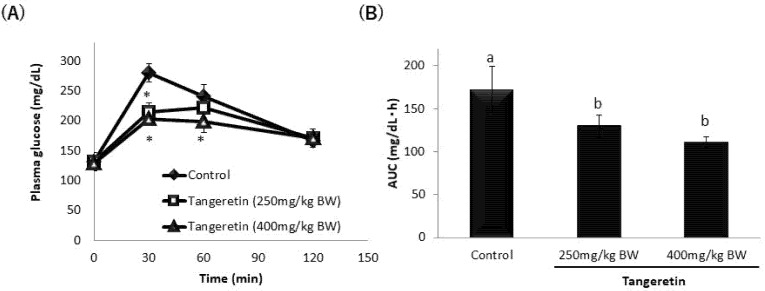
Effect of tangeretin on plasma glucose levels after oral glucose administration in ICR mice. ICR mice fasted overnight were orally administered 1 g glucose/kg body weight (BW) (20% glucose solution) along with or without 250 mg or 400 mg tangeretin/kg BW, and blood was drawn at 0, 30, 60, and 120 min. The plasma glucose concentration was measured (**A**), and the AUC under each experimental condition was calculated based on the values obtained (**B**). The values are expressed in terms of mean ± standard deviation of mean (*n* = 6); (**A**) * *p* < 0.05 vs. the control value (Dunnett’s test). (**B**) The values indicated by the different characters are significantly different from each other (Tukey’s test; *p* < 0.05).

**Figure 10:**
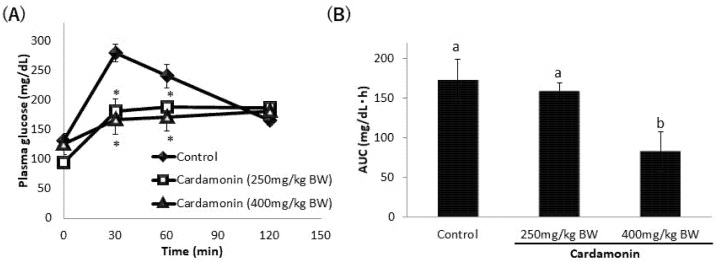
Effect of cardamonin on plasma glucose levels after oral glucose administration in ICR mice. ICR mice fasted overnight were orally administered 1 g glucose/kg body weight (BW) (20% glucose solution) along with or without 250 mg or 400 mg cardamonin/kg BW, and blood was drawn at 0, 30, 60, and 120 min. The plasma glucose concentration was measured (**A**), and the AUC under each experimental condition was calculated based on the values obtained (**B**). The values are expressed in terms of mean ± standard deviation of mean (*n* = 6); (**A**) * *p* < 0.05 vs. the control value (Dunnett’s test). (**B**) The values indicated by the different characters are significantly different from each other (Tukey’s test; *p* < 0.05).

To confirm the inhibitory effect of tangeretin and cardamonin on SGLT1 activity in vivo, we performed OGTTs in ICR mice. The oral administration of tangeretin significantly suppressed the rapid spike in the blood glucose levels (Figure 9A). The AUC value also decreased upon the co-administration of tangeretin (Figure 9B). Cardamonin suppressed the rapid spike in the blood glucose levels and AUC values (Figure 10A,B). These findings suggested that tangeretin and cardamonin suppress blood glucose spikes in vivo.

## 4. Discussion

In the present study, we established a stable hSGLT1-expressing CHO cell line and attempted to identify phytochemicals that could inhibit SGLT1 activity. We found that two phytochemicals, tangeretin and cardamonin, significantly inhibited SGLT1 activity via different mechanisms. OGTTs were performed using the mice, which further confirmed that tangeretin and cardamonin significantly suppressed rapid blood glucose spikes, suggesting the in vivo SGLT1 inhibitory activity of the compounds.

In this study, we found that tangeretin and nobiletin markedly inhibited SGLT1 activity. Tangeretin and nobiletin are both flavonoids present in citrus fruits such as *Citrus unshiu*, *C. depressa*, *C. tangerina*, and *C. hassaku*. As the hydroxyl groups of these compounds are methoxylated, these are also referred to as methoxyflavonoids. Methoxyflavonoids contain a methoxy group in the flavone backbone, and therefore, exhibit high hydrophobicity. In recent years, various functional properties of methoxyflavonoids present in citrus fruits have been reported, including anti-inflammatory, anti-tumor, and anti-obesity effects [20,21,22]. *C. unshiu* contains various bioactive components, such as auraptene, β-cryptoxanthin, and limonine. Many methoxyflavonoids, such as tangeretin, nobiletin, hesperetin, and sinensetin, are also present in the form of glycosides and contribute to the functionality of *C. unshiu*. However, at least based on our findings, the relationship between SGLT1 activity and tangeretin or nobiletin remains unreported, and the SGLT1 inhibitory activities of tangeretin and nobiletin may be considered novel.

Interestingly, even though tangeretin and nobiletin exhibited significant inhibitory activity against SGLT1, sinensetin did not exhibit an inhibitory effect, as shown in Figure 6. However, tangeretin, nobiletin, and sinensetin significantly inhibited fructose uptake via GLUT5 in Caco-2 cells [16]. Tangeretin and sinensetin both possess a flavone structure with five methoxy groups, and nobiletin possesses a flavone structure with six methoxy groups. The structures of the compounds are considerably similar, but only sinensetin failed to exhibit inhibitory activity. Therefore, these results clearly indicate that the inhibitory effects of the three methoxyflavonoids against SGLT1 and GLUT5 are different. Based on these findings, we assumed that the methoxy group at position 8 of the A ring, which is common only to tangeretin and nobiletin, is essential for SGLT1 inhibition, but not for GLUT5 inhibition. Further, the analysis of various tangeretin-related chemical compounds is necessary for the thorough evaluation of the structure–activity relationship between methoxyflavonoids and SGLT1.

We also confirmed that cardamonin inhibits SGLT1 activity. Cardamonin is a type of methoxychalcone that is abundantly present in the seeds of *Alpinia katsumadai* (ginger plant) and the rhizome of *Boesenbergia pandurata* (Chinese bamboo shoot). In recent years, several functional polyphenols have been identified in plants native to Southeast Asia, and among these, cardamonin has been reported to exhibit several bioactive properties, including antitumor, anti-mutagenic, anti-inflammatory, and antioxidant properties [23,24,25]. With respect to its action on glucose transporters, cardamonin was reported to increase glucose uptake in L6 myotubular cells by promoting GLUT4 translocation to the plasma membrane [26]. However, to the best of our knowledge, there have been no reports on the effect of cardamonin on intestinal glucose absorption. The SGLT1 inhibitory activity of cardamonin is considerably novel. The effect of structural analogues of cardamonin on SGLT1 activity was examined, and flavokawain B, which has the same methoxychalcone structure as cardamonin, was also found to exhibit significant inhibitory activity against SGLT1. In addition, naringenin chalcone also exhibited strong inhibitory activity, whereas alpinetin and naringenin, which possess flavanone structures, exhibited limited inhibitory activity (Figure 7). These findings suggested that the methoxychalcone structure is important for the inhibition of SGLT1 activity. We intend to examine the effects of other compounds with a methoxychalcone structure on SGLT1 activity.

Kinetic analysis was performed to analyze the characteristics of the SGLT1 inhibitory activity of the phytochemicals (Figure 8). Cardamonin did not significantly alter the K_m_ value, but decreased the V_max_ values to approximately three-fourths of the original value. Conversely, tangeretin increased the K_m_ value by approximately two-fold and decreased the V_max_ value by approximately half (Figure 8). The results suggested that cardamonin exerted a non-competitive inhibitory effect, since cardamonin significantly reduced the V_max_ without significantly altering the K_m_ value. In the case of non-competitive inhibition, the inhibitor acts at a site different from the active site to inhibit transporter activity. Glucose transporters are considered to transport substrates as glucose-gated channels. Cardamonin was found to act at a site different from the substrate recognition site of SGLT1. Conversely, tangeretin considerably increased the Km value and decreased the Vmax value, suggesting that it exhibits both competitive and non-competitive inhibitory activity. Thorough analysis based on the crystal structure of human SGLT1 may help understand the inhibitory mechanism at the molecular level using docking simulation.

Reportedly, tiliroside, present in rosehip extract, inhibited the increase in blood glucose level caused by simultaneous administration of glucose in ICR mice [27,28]. Additionally, the long-term administration of nobiletin was reported to improve postprandial blood glucose levels in diabetic mouse models [29,30,31]. Furthermore, although the glucose transporter in intestinal epithelial cells was not targeted, the oral administration of *Ashitaba* extract, which is known to promote glucose uptake in skeletal muscles, in ICR mice was reported to significantly suppress postprandial blood glucose levels [32]. In the present study, the OGTTs performed in ICR mice showed that 250 mg of tangeretin significantly suppressed the increase in blood glucose 30 min after administration, 400 mg of tangeretin significantly suppressed the increase in blood glucose 30 and 60 min after administration, and both 250 mg and 400 mg of tangeretin significantly decreased the AUC. Meanwhile, both 250 mg and 400 mg of cardamonin significantly suppressed the increase in blood glucose levels at 30 and 60 min after administration, whereas the blood glucose level at 120 min after administration was almost equal to that in the control group. Furthermore, the AUC decreased significantly upon the administration of 400 mg of cardamonin, suggesting that cardamonin delayed the blood glucose spike.

Further, to confirm the in vivo inhibition of SGLT1 by these phytochemicals, intraperitoneal glucose tolerance tests should be conducted. When administered intraperitoneally rather than orally, glucose enters the bloodstream of the body via the portal vein without being absorbed from the intestinal tract. Therefore, it can be determined whether the inhibitory effects of phytochemicals on elevated blood glucose levels is attributed to the inhibition of intestinal glucose absorption. For example, a significant decrease in the blood glucose level and AUC was noted after the oral administration of tiliroside, whereas there was no decrease in the blood glucose level and AUC after intraperitoneal administration of tiliroside, confirming that tiliroside suppressed the increase in blood glucose levels by inhibiting intestinal epithelial glucose transporters [27].

## 5. Conclusions

In the present study, we developed a stable hSGLT1-expressing CHO cell line and used it to identify phytochemicals that inhibit SGLT1 activity using a simple in vitro method. We found tangeretin and cardamonin to be SGLT1 inhibitory compounds and confirmed their novelty. Further, these two compounds effectively prevented rapid blood glucose spikes in mice in vivo. Our findings indicate the potential of these compounds as functional food components with inhibitory effects on blood glucose elevation. Further, the developed cell line can be used to identify other functional food components that can inhibit SGLT1 activity.

## Data Availability

The data that support the findings of this study are available from the corresponding author, H.S., upon reasonable request.

## Appendix A

**Table A1:** Phytochemicals used in this experiment.

***Compounds***	***Company***
Myricetin	Sigma-Aldrich
Morin	Sigma-Aldrich
Puerarin	Sigma-Aldrich
Rutin	Wako
Fisetin	Sigma-Aldrich
Kaempherol	Extrasynthese
Quercitrin	Tokyo Chemical Industry
Quercetin	Tokyo Chemical Industry
Genistin	Fujicco
Diosmin	Sigma-Aldrich
Ginkgolide J	Tama Biochemical
Ginkgolide B	Tama Biochemical
Baicalein	Wako
Apigenin	Sigma-Aldrich
Flavonol	Sigma-Aldrich
Flavanone	Wako
Flavone	Sigma-Aldrich
Hesperetin	Sigma-Aldrich
Galangin	Sigma-Aldrich
Genistein	Sigma-Aldrich
Tangeretein	Wako
Daidzein	Sigma-Aldrich
Naringin	Sigma-Aldrich
Naringenin	Sigma-Aldrich
Flavone	Sigma-Aldrich
Daidzin	Wako
Equol	LC laboratories
Nobiletin	Wako
Glycitin	Wako
Glycitein	Wako
Caffeine	Sigma-Aldrich
Sinensetin	Wako
Rosmaric acid	Wako
Resveratrol	Wako
Xanthohumol	Tokyo Chemical Industry
4-Hydroxyderricin	Medchemexpress
Xanthoangerol	Medchemexpress
Cardamonin	Medchemexpress
Isoindigo	Cayman
Indirubin	Tokyo Chemical Industry
Indigo	Wako
Quercetin-3,4’-di-O-Glucoside	Extrasynthese
Isoquercitrin	Cayman
Spireoside	Sigma-Aldrich
Chrysin	Tokyo Chemical Industry
Pinostrobin	Sigma-Aldrich
Pinocembrin	Wako
Alpinetin	Selleck
Monoglucosyl hesperidin	Wako
Hesperidin	Tokyo Chemical Industry
